# Genome-Wide Association Study to Identify Possible Candidate Genes of Snap Bean Leaf and Pod Color

**DOI:** 10.3390/genes14122234

**Published:** 2023-12-18

**Authors:** Burcu Celebioglu, John P. Hart, Timothy Porch, Phillip Griffiths, James R. Myers

**Affiliations:** 1 Department of Horticulture, Oregon State University, 4017 Ag & Life Science Bldg., Corvallis, OR 97331, USA; celebiob@oregonstate.edu; 2 USDA-ARS, Tropical Agriculture Research Station (TARS), 2200 P. A. Campos Ave., Suite 201, Mayagüez, PR 00680, USA; jph248@cornell.edu (J.P.H.); timothy.porch@usda.gov (T.P.); 3 School of Integrated Plant Sciences, Horticulture Section, Cornell Agritech, 635 W. North St., Geneva, NY 14456, USA; pdg8@cornell.edu

**Keywords:** blink, chroma, colorimeter, CURT1, edible-podded bean, GBS, GWAS, hue, KAC1, leaf, *Phaseolus vulgaris*, pod, common bean, SNP

## Abstract

Color can be an indicator of plant health, quality, and productivity, and is useful to researchers to understand plant nutritional content in their studies. Color may be related to chlorophyll content and photosynthetic activity and provides information for those studying diseases and mineral nutrition because every nutrient deficiency and many diseases produce symptoms that affect color. In order to identify significant loci related to both leaf and pod color in a snap bean (*Phaseolus vulgaris* L.) diversity panel, a genome-wide association study (GWAS) was carried out. Leaf color in one and pod traits in multiple environments were characterized using a colorimeter. L*a*b* color data were recorded and used to calculate chroma (C*) and hue angle (H°). Leaves were evaluated at three positions (lower, middle, and upper) in the canopy and both pod exterior and interior colors were obtained. GWAS was conducted using two reference genomes that represent the Andean (G19833) and Middle American (5-593) domestication centers. Narrow sense heritabilities were calculated using the mixed linear model (MLM) method in genome association and prediction integrated tool (GAPIT), and significant single nucleotide polymorphisms (SNPs) for each color parameter were obtained using the Bayesian-information and linkage-disequilibrium iteratively nested keyway (BLINK) GWAS model with two principal components (PCAs). In comparison to pod color traits, narrow sense heritabilities of leaf traits were low and similar for both reference genomes. Generally, narrow sense heritability for all traits was highest in the lower, followed by middle, and then upper leaf positions. Heritability for both pod interior and exterior color traits was higher using the G19833 reference genome compared to 5-593 when evaluated by year and means across years. Forty-five significant SNPs associated with leaf traits and 872 associated with pods, totaling 917 significant SNPs were identified. Only one SNP was found in common for both leaf and pod traits on Pv03 in the 5-593 reference genome. One-hundred thirteen significant SNPs, 30 in leaves and 83 in pods had phenotypic variation explained (PVE) of 10% or greater. Fourteen SNPs (four from G19833 and ten from 5-593) with ≥10 PVE%, large SNP effect, and largest *p*-value for L* and H° pod exterior was identified on Pv01, Pv02, Pv03, and Pv08. More SNPs were associated with pod traits than with leaf traits. The pod interior did not exhibit colors produced by anthocyanins or flavonols which allowed the differentiation of potential candidate genes associated with chloroplast and photosynthetic activity compared to the pod exterior where candidate genes related to both flavonoids and photosynthesis affected color. Several SNPs were associated with known qualitative genes including the wax pod locus (*y*), persistent color (*pc*), purple pods (*V*), and two genes expressed in seeds but not previously reported to affect other plant tissues (*B* and *J*). An evaluation of significant SNPs within annotated genes found a number, within a 200 kb window, involved in both flavonoid and photosynthetic biosynthetic pathways.

## 1. Introduction

Color has many roles in plants, from abiotic and biotic stress response to pollinator attraction. For crop plants, color may be characteristic of certain cultivars and market classes and is a primary quality factor because it can be an indicator of plant health and nutrient content. At each development stage, deviations in color may signal differences in photoassimilation and nutrient accumulation. Commission Internationale de l’Éclairage (CIE) L*a*b* is the most common method used to determine color in agricultural and food research [1].

Snap bean is a vegetable type of common bean (*P. vulgaris* L.) that is high in certain vitamins and minerals not found in mature seeds. To date, while flower, seed, and pod colors have been studied in bean genetic research, there has been little investigation of leaf colors. This may be because leaf color in snap beans shows relatively little variation, but pod color is a key characteristic and shows substantial variation in shades of green between accessions, as well as wax types with yellow pods and specialty types with purple pods.

Chlorophyll content directly correlates to the greenness of tissues, anthocyanins and flavonols may produce colors ranging from yellow to brown to blue and red to purple, and carotenoids may be responsible for some yellow and orange hues. Color in seeds of common beans is usually associated with flavonoids and they may affect human health because of their antioxidant and anticarcinogenic characteristics [2]. Genes in the phenylpropanoid (flavonoid) pathway, including down-stream flavonols and anthocyanins, have a crucial role in processes like disease resistance, nodulation (biological N_2_ fixation), and UV protection, which determine how plants interact with their environments. In an apparent paradox, snap bean breeders select for the absence of flavonoid pigments because their presence affects the quality of processed snap bean pods. While the pods of most accessions with white flower and seed color are various shades of green, the pod color of accessions with colored flower and seed color may have red or purple hues. Snap beans are selected for white seed because canned and frozen snap bean pods may be discolored in the product when water-soluble flavonoids leach from colored seeds [3]. However, for bean pods intended for fresh markets, seed colors can also be colored. High levels of anthocyanins and flavonols are often associated with cultivars with colorful seed and flower colors.

Some of the genes controlling flower, seed, and pod color in common bean (Table 1) have multiple allelic complexes [4]. These may affect flavonoid, chlorophyll, and carotenoid biosynthetic pathways. The dominant *P* (*pigment*) gene allows other genes to express color in various plant parts, while in plants with the recessive gene (*p*) these tissues lack flavonoid pigments. The recessive homozygous (*p*^gri^) allele allows weak expression which results in a faded color in seeds and attenuated flower color. Color changes in other plant parts have not been documented with this allele but it is likely that purple color in stems and pods would also be attenuated. The *P* gene with *T* gives a completely colored seed coat color while recessive *t* gives a partial seed coat color [5] and changes flower color from pigmented to white. Its effect on stems and pods has not been described. The *T* gene is epistatic to *Bip* and *Z* genes [6]. These genes modify patterning induced by *t* but have not been reported to affect plant tissues other than seeds. The *V* gene gives purple or violet color to flowers with pleiotropic effects on seeds, leaves, stems, and pods, while recessive homozygous *v* gives white and *v*^lae^ gives pink flower color [5]. Seed coats with the dominant *V* genotype contain anthocyanin pigments, while seeds with recessive *v* are unpigmented, or have flavonol pigments if *B* or *G* are present [7,8]. *V*, *G*, and *B* interact with each other and give various seed coat colors from yellow to black. For example, dominant *V* with dominant *B* gene conditions black seeded beans [9]. While *V* can affect various plant parts, changes in vegetation and pod color due to *G* and *B* have not been reported. *C* is a complex locus that regulates the patterning of other color genes, producing striping and mottling on seeds and pods [5]. *C* is hypostatic to *P*, *B*, *G*, and/or *V* and is tightly linked to the dominant red *R* gene. The *C* locus may also affect the expression of *Prp* genes. *Prp* is multiallelic and controls purple color in pods. It may also affect other tissues of the bean plant as typified by the *c*^u^*Prp*^i^ gene combination that intensifies anthocyanin expression [10]. *V* with *Prp* gives purple color to the pod but the intensity of color is variable [5]. *B* conditions greenish (grey) seed coat color and it is tightly linked with the *I* gene that provides resistance to bean common mosaic virus [5,11]. *J* or the *joker* gene conditions shiny seed coats that darken as the seeds age whereas recessive *j* attenuates the expression of other seed coat colors and produces a dull seed coat that does not darken with age. Expression in plant parts other than seed has not been reported. The recessive *y* gene with dominant *Arg* produces yellow (wax) color pods, while the dominant *Y* gene with recessive *arg* gives greenish gray (silver) color pods, and dominant genes (*Y*-*Arg*-) condition green color pods [12]. When both genes are recessive, pods are white in color. The *persistent color* (*pc*) gene is a member of the Stay green gene family and conditions a pale green seed testa. Fresh pods are uniformly green and remain green because *pc* prevents chlorophyll loss and senescence in the plant tissues [13]. The *ace* (*acera*) gene in its recessive form produces a shiny pod and may act through the reduction of epicuticular wax and pubescence. It is linked to *V* on Pv06. Some color genes have been mapped and/or candidate genes proposed, but the majority remain to be identified (Table 1).

A GWAS is an approach to mapping and characterizing quantitative trait loci (QTL) in populations of related individuals. There are several software packages such as trait analysis by association, evolution and linkage (TASSEL), PLINK, and GAPIT for conducting GWAS. GAPIT (version 3) supplies one of the simplest ways to analyze data using multiple statistical models like MLM, Blink, and Fixed and random model Circulating Probability Unification (FarmCPU) using fixed and random effects. BLINK is a modified form of the FarmCPU. FarmCPU uses the maximum likelihood method with kinship as a random effect, while kinship in the Bayesian Information Content (BIC) in BLINK is a fixed effect. In BLINK, significant markers based on linkage disequilibrium (LD) are subject to a process of elimination until no significant markers remain [22].

The objective of this study was to use genome-wide association studies to identify SNPs associated with genes controlling leaf and pod color, determine the genetic architecture of tissue color traits, and identify potential candidate genes for color. 

## 2. Materials and Methods

### 2.1. Plant Material

The SnAP (Snap bean Association Panel) consisting of 378 accessions, was used in this study. The panel includes accessions with determinate bush (CIAT classification type I) growth habits, but a few were half-runners (type III), and another subset had an indeterminate (type IV) climbing growth habit. The accessions represent different market classes, such as Blue Lake, European small sieves, Refugee and Romano beans, and yellow (wax) beans of American or European origin [23]. 

The accessions were planted in the Oregon State University greenhouse in the fall and winter of 2018–2019 and at the Oregon State University Vegetable Research Farm (lat. 44.573778, long. −123.236750) in the summer of 2019 and 2020. In both study areas, the population was split into five blocks based on flowering date, and the blocks were planted in succession with 2–5 days in between to spread out the data collection workload. Five seeds of each accession were sown in each pot for greenhouse trials while sixty seeds were used in each plot for field trials. In the greenhouse, the 10 cm dia. pots were supplemented with 3.5 g of Osmocote 14-14-14 NPK fertilizer in soilless media (Sungro Horticulture, Agawam, MA, USA), and bamboo stakes were used to support each plant when necessary. The soil of Oregon State University Vegetable Research Farm is a Chehalis silty clay loam (fine-silty, mixed, superactive, mesic Cumulic Ultic Haploxeroll). Before planting, 16-16-16 NPK fertilizer was banded in the row, and the seeds were sown approximately 2.5 cm deep in rows 5 m long and 76 cm apart with a hand-propelled belt planter. The pole beans (type IV) were trellised using metal wires hung on T-posts, but bush beans (type I) did not need support in the field. Plants were irrigated as needed in the greenhouse and by overhead sprinklers in the field applying about 2.5 cm at least once a week.

### 2.2. Data Acquisition

Color was measured using a colorimeter (Minolta BC-10; Konica Minolta Sensing Americas, Ramsey, NJ, USA), which recorded color parameters using the CIE L*a*b* system [1]. The white point was calibrated for each user session using a white card that the manufacturer provided. Color measurements were conducted on 376 accessions for leaf color in 2019 and 378 accessions for pod color in 2019 and 2020. The leaf color was measured in the greenhouse using three plants per accession with measurements taken at three positions (lower, middle, and upper) in the canopy. The leaves at each position of each plant were measured three times to produce a mean value at each leaf position. Leaf measurements were taken when the plants began to flower. The pod color was measured in the field and five pods were randomly collected from each accession at processing harvest maturity (this stage corresponds to 50% 1–4 sieve size or seeds 1 cm in length [3]). After the pods were cut in half and the seeds removed, both external and internal colors were measured on each half. For phenotypic analysis, lightness (L*) chroma (C*), and hue (H°) were calculated from a* and b* parameters as:

C*_ab_ = [a*^2^ + b*^2^]^1/2^

H° = tan^−1^ (b*/a*)

The genotypic data were acquired from the Cornell University of Biotechnology Genomic Diversity Facility [24]. The SnAP diversity panel was subjected to genotype by sequencing (GBS) and was initially aligned to the G19833 (v1.1) reference genome. It was subsequently realigned to the G19833 (v 2.1) and 5-593 (v1.1) reference genomes. HapMap files of the SnAP aligned to the two reference genomes were produced by Alvaro Soler Garzon of the Miklas Lab, USDA-ARS, Prosser, WA. Alignment and imputation methods are described by Saballos et al. [23]. After 0.01 minor allele frequency (MAF) filtering, 25,472 SNPs were generated with G19833 (v2.1), and 34,442 SNPs were generated with 5-593 (v1.1) reference genomes. G19833 represents the Andean center of domestication (race Nueva Granada) and 5-593 represents the Middle American center of domestication (race Mesoamerica).

### 2.3. Genome-Wide Association Study

The leaf color with three positions and pod exterior and interior colors were used for GWAS analysis. For leaf color data, greenhouse measurements were used in 2019, whereas two years of pod color data (2019 and 2020) and combined data (means) for both years were performed for pod color analysis using the color parameters L*, a*, b*, C*, and H°.

Three different models (one single locus and two multiple loci) were tested in the GAPIT package in R ver. 4.2.2 and five principal components (PCs) from 0 to 4 were utilized for each model. The models used were MLM, BLINK, and FarmCPU. Based on QQ plots and scatter graphs of PCs, the BLINK model with two PCs was determined to produce the best fit for the data for GWAS analysis. SNPs were filtered using an MAF of 0.05 with a Bonferroni significance threshold of 0.01. GWAS was conducted separately for the SNP alignments of the two reference genomes. Narrow sense heritability estimates of traits were produced using the MLM model in GAPIT. 

### 2.4. Candidate Gene Research

Once all significant SNP positions were identified, candidate gene searches were carried out in a 100 kb window upstream and downstream of SNPs associated with each trait and for both reference genomes. Potential candidate genes identified in the G19833 reference genome were examined in Phytozome (https://phytozome-next.jgi.doe.gov/ (accessed on 5 July 2023)) to identify those with the highest expression in plant parts based on levels and traits (leaves and young trifoliolates for leaf traits and in young pods, green mature pods, flower, and flower buds for pod traits). However, there was no comparable RNA-seq data available for the 5-593 reference genome. For gene models located within 100 kb windows of significant SNPs, Arabidopsis genes corresponding to the *Phaseolus* gene models were identified. These were investigated in Tair (https://www.arabidopsis.org/ (accessed on 5 July 2023)). The final set of candidate genes was chosen based on tissue expression (leaves and pods), candidate gene location (chloroplast or thylakoids for example), and association with various biosynthetic pathways (e.g., chloroplast development, photosynthesis pathway, wax biosynthesis, UV-B light protection, and flavonoid biosynthesis). For *Phaseolus* gene models within SNP windows without a corresponding Arabidopsis gene model, protein sequences were blasted (https://blast.ncbi.nlm.nih.gov/Blast.cgi (accessed on 5 July 2023)) and after sorting, those with the lowest e-value and more than 70% percent identity were selected, and evaluated for function.

## 3. Results

### 3.1. Narrow-Sense Heritability

Trait narrow-sense heritability (*h*^2^) was obtained from GWAS against two reference genomes using an MLM model in GAPIT (Table 2). The *h*^2^ for leaf positions tended to be low, about 25–40% for all traits, except upper leaf position for a*, which was very low (~14%). In general, upper leaf position *h*^2^ was lower than other positions, but the differences were small. When compared to broad sense heritability (*H*), *h*^2^ was about half as large [25]. For both reference genomes, leaf *h*^2^ was significantly lower compared to *h*^2^ for pod color traits, which ranged from moderate to high (56–85%). Unlike leaf *h*^2^, pod *h*^2^ was similar to or higher than *H* for corresponding traits (Table 4 in [25]). Pod interior color *h*^2^ was highest for H°, L*, and then C*, and exterior *h*^2^ were similar across all traits (Table 2). Pod interior heritabilities were generally higher than exterior with the exception of b* and C*, where interior heritabilities were lower than exterior. 

### 3.2. Significant SNPs

#### 3.2.1. Overall Description of Significant SNPs 

A total of 917 significant SNPs were identified using a Bonferroni threshold of 0.01: 45 for leaves (19 from G19833 and 26 from 5-593) and 872 for pods (337 from G19833 and 535 from 5-593). While 70 of these SNPs lay outside of the gene coding regions (highlighted in blue, Tables S1 and S2), the other SNPs were inside the gene coding regions. In windows 100 kb upstream and downstream of significant SNPs across environments and traits, there were 292 unique SNPs: 148 from G19833 and 144 from 5-593 reference genomes (Table S2). When considered by exact SNP position, the numbers of unique SNPs were 210 for G19833 and 361 for 5-593 (Table S1). One-hundred thirteen of all SNPs had phenotypic variance explained (PVE) ≥ 10%, which included 30 SNPs for leaf traits (16 from G19833 and 14 from 5-593) and 83 SNPs for pod traits (40 from G19833 and 43 from 5-593) (Table 3, Table 4 and Table 5). Sixty percent of significant SNPs for pods were identified in single years (nine from 2019 and 15 from 2020), and 40% (16 SNPs) were observed in both years for the G19833 reference genome. Proportions were similar for the 5-593 reference genome: 67% from single years (12 from 2019 and 17 from 2020), and 33% (14 SNPs) from combined years. SNP effects of leaf and pod interior parameters were lower than pod exterior parameters, while probabilities (−log_10_(*p*)) were lower in the leaves than for pod color parameters (Table 3, Table 4 and Table 5).

#### 3.2.2. SNPs Associated with More than One Tissue

Two SNPs, one from G19833 (Pv02 position 807,178 bp) and one from 5-593 reference (Pv02 position 1,604,407 bp), were associated with both pod interior and exterior color parameters (Table 4 and Table 5 and Table S1). Among unique SNPs in the G19833 genome, one was associated with different plant tissues (leaves vs. pods) and nine were found in both pod interior and exterior. Similarly, in the 5-593 genome, 11 SNPs were associated with more than one plant part, with one found in leaves and pods and 10 found in pod interior and exterior.

SNPs in common for both leaves and pods were found on Pv09 and Pv10 in the G19833 genome and on Pv03 and Pv10 mapped to 5-593 (Table S1). The SNP on Pv03 had the same position for both tissues but other SNPs were also found within 100 kb of this SNP. 

#### 3.2.3. SNPs Associated with More than One Color Trait 

Forty-eight SNPs in the G19833 genome and 54 in 5-593 were associated with more than one trait. Some autocorrelation among traits was expected. This was mainly for a* and b* with C* and H° because the former two variables are used in calculations of the latter two, and this was found in many cases where the same SNP was associated with more than one parameter. L* does not share a mathematical relationship with the other parameters, but less commonly, the same SNP was associated with this and other color parameters. Thirteen SNPs shared uncorrelated traits (usually L* with a* or C*) in the G19833 genome and 20 in the 5-593. 

#### 3.2.4. SNPs Associated with Leaves 

Forty-five SNPs were associated with color traits in leaves, with 13 for the lower leaf position, 20 for the middle leaf position, and 12 SNPs for the upper leaf position. SNPs were associated with b* (middle leaf position) on Pv01, a* (middle) on Pv02, L* (middle) and b*, C*, and H° (upper) on Pv09, and b* (lower and middle) on Pv10 across the two genomes (Table S1). PVEs ranged from 11 to 57% and generally were higher in leaves compared with exterior and interior pod color traits (Table 3, Table 4 and Table 5). Some leaf color parameters like L* (middle leaf position), a* (middle), b* (lower, middle, and upper), C* (upper), and H° (upper) with ≥10 PVE were found mostly on the same chromosome (Table 3). 

SNPs with a large effect for a* (middle leaf position) were found on Pv02 in both reference genomes and of similar magnitude (30.7% for G19833 and 28.9% for the 5-593 genome). For a* (upper leaf position), PVE was 33.6% on Pv01 in G19833 and 35.1% on Pv09 in 5-593 (Table 3). 

The PVE and SNP effect of b* (lower leaf position) was relatively large in the 5-593 reference genome (Pv01, 24.7% and 2.4, respectively), while a significant SNP for the same trait in the G19833 (Pv01, 20.4% and −2.1) had a similar magnitude (with reversed sign for SNP effect) on the same chromosome (Table 3). A similar pattern was observed for b* (upper leaf position) with larger PVE and SNP effect for G19833 (Pv09, 55.5% and −3.1) than 5-593 (Pv09, 33.6% and 3.0). 

The PVE and *p*-value for C* (upper) on Pv09 were similar in magnitude in 5-593 (55.1% and 10.34, respectively) compared to G19833 (54.8%, 9.42). 

Even though H° of middle and upper leaf positions were on Pv09 in both reference genomes, the middle leaf position was only in the 5-593, and the upper leaf position had high PVE in the 5-593 (57%) and similar to that found in G19833 genomes (56.9%) (Table 3).

#### 3.2.5. SNPs Associated with Pod Exterior Color 

**Figure 1:**
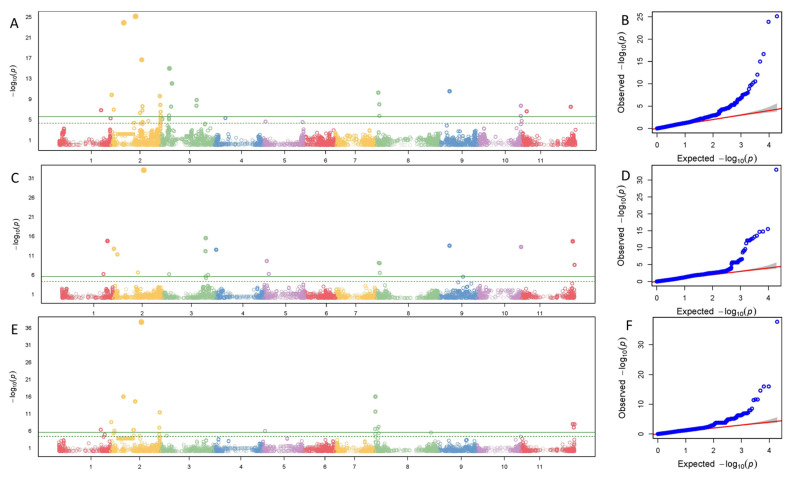
Manhattan and Quartile-quartile (QQ) plots of hue angle pod exterior color using the G19833 reference genome with the snap bean association panel (SnAP). (**A**) Manhattan plot of the trait observed in 2019 with the 11 chromosomes on the x axis and the *p*-value (−log_10_(*p*)) on the y axis. Solid green line is Bonferroni cutoff for significant SNPs and dashed line is the false discovery rate (FDR) threshold. (**B**) QQ plot corresponding to the Manhattan plot in (**A**). (**C**) Manhattan plot of the trait in 2020. (**D**) QQ plot of the Manhattan plot in (**C**). (**E**) Manhattan plot combined over years. (**F**) QQ plot of the Manhattan plot in (**E**).

**Figure 2:**
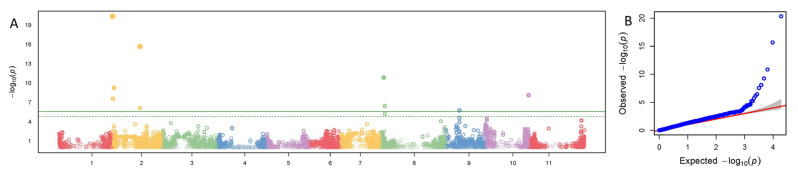
Manhattan and Quartile-quartile (QQ) plot of L* pod exterior color using the G19833 reference genome with the snap bean association panel (SnAP). (**A**) Manhattan plot for combined years with the 11 chromosomes on the x axis and the *p*-value (−log_10_(*p*)) on the y axis. Solid green line is Bonferroni cutoff for significant SNPs and dashed line is false discovery rate (FDR) threshold. (**B**) QQ plot corresponding to the Manhattan plot in (**A**).

Significant SNPs with ≥10 PVE for pod exterior were on Pv01, Pv02, Pv03, and Pv04 in both reference genomes, and on Pv08, Pv09, Pv010, and Pv011 in just the G19833 genome (Table 4). PVE ranged from 10.8 to 29.9 for a* in the G19833 and from 13.5 to 36.7 in the 5-593 genomes. For b* PVE, it ranged from 10.3 to 45.2 in G19833 and between 15.7 and 29.6 in 5-593. PVE of L* ranged from 11.1 to 25.8 on G19833 and 10.2 to 19.6 for 5-593 genomes. C* and H° had a wider PVE range in the G19833 (11.9 to 26.4 and 10.9 to 32.0, respectively) than 5-593 (13.8 to 24.6 and 11.3 to 29.3) (Figure 1, Table 4). One SNP associated with L* with high PVE had also a large effect (11.1), and *p*-value at 27.7 Mb on Pv02 in the G19833 genome (Figure 2, Table 4).

Fourteen SNPs (four from G19833 and ten from 5-593) that had a combination of PVE ≥ 10%, large SNP effects, and highly significant *p*-values were expressed for L* and H° pod exterior (Table 6). Although SNPs from all tissue types were evaluated, only those for external pod color met these criteria. SNPs for L* were found on Pv02 in the G19833, and those for H° were found on Pv03 and Pv08 in the G19833, and Pv01, Pv02, and Pv03 in the 5-593 (Table 4 and Table 6). The SNP effects were evenly divided (seven negative and seven positive) in the direction of the effect. 

#### 3.2.6. SNPs Associated with Pod Interior Color

There were 25 significant SNPs associated with various traits and environments (15 from G19833 and 10 from 5-593) with high PVE for pod interior (Table 5). The same SNP in the 5-593 reference genome on Pv02 was associated with L*, a*, H°, and C* in single- and two-year environments. Those SNPs with the highest *p*-values from both reference genomes were on Pv02. L* PVE was similar in the 5-593 (13.3–20.9) compared to the G19833 genome (14.0–19.1). 

#### 3.2.7. Potential Candidate Genes Associated with SNPs

The greatest number of SNPs were observed on Pv02 and many of these were associated with genes affecting pod and leaf color. Potential candidate genes associated with SNPs on Pv02 include Phvul.002G007200 (Pv5-593.02G006900) associated with an SNP at 790,825 bp (1,586,653 bp) that may affect the expression of *y*, Pv5-593.02G134200 and Pv5-593.02G134900 both associated with an SNP at 29,034,303 bp could be associated with chlorophyll production of chloroplast movement, and Phvul.002G153100 (Pv5-593.02G149800) which colocalized with SNPs at 30,590,620 bp and 31,120,266 bp, respectively may be a candidate for *pc* (Table 1 and Table 6 and Table S1). Phvul.002G319500, Phvul.002G319600, and Phvul.002G319800 associated with a SNP at 48,664,812 in G419833 are possible candidates for *B* (Table 1 and Table S1). On other chromosomes, Phvul.007G103400 is a candidate associated with a SNP at 11,974,591 bp for L* pod interior in G19833 and it is a homolog of Glyma.20G187000 (*Yl*) in *Glycine max*. Phvul.010G132400 and Phvul.010G132500 associated with a SNP at 41,403,581 bp on Pv10 in the G19833 reference genome may be associated with *j* (Table 1 and Table S1). Further details on these and other candidate genes are detailed in the discussion.

## 4. Discussion

Changes in color may represent different plant responses in different tissues to factors such as disease, nutrient deficiency, drought or salt stress, nitrogen fixation, hormones like ethylene, light intensity, heat, chlorophyll biosynthesis, and photosynthetic rate. Many genes regulate the flavanol and anthocyanin biosynthesis pathway that controls color in common beans. While most accessions in the SnAP have white flower color, some accessions did have pink and purple flower colors. Most have green stem color, but some pole accessions have purple stems. Accessions have a range of seed coat colors from completely white, to white with dark hilum, to greenish-white, to red, to black. Pod colors are mostly green from light to dark, yellow, and purple, but some pods were green with purple striping. Many seed coat color and pattern genes require *P* to be expressed [4] and because the majority of accessions in the SnAP have recessive *p*, various color genes may be present in the diversity panel that do not have a visible phenotype in seeds and flowers. However, these genes may still have phenotypic effects in leaves and pods that have not been previously documented. Potential examples of this are described below for the *B* and *j* loci.

### 4.1. Candidate Genes Associated with the Flavonoid Biosynthetic Pathway

The *B* gene, designated as the precursor of any compound preceding dihydrokaempferol in the flavonoid color pathway, is on Pv02 [8]. The gene position was located at 48,634,623–48,634,743 bp associated with the SNP ss715645998 [14] (Table 1). Three candidate genes (Phvul.002G319500, Phvul.002G319600, and Phvul.002G319800 in G19833 genome) in our study at a distance of about 65–90 kb to an SNP at 48,664,812 bp are involved in the anthocyanin pathway and are potential candidates for *B* gene (Table S1) [26,27,28].

Phvul.002G317000 was found within a 100 kb window of an SNP at 48,416,815 bp associated with C* pod exterior in G19833. It is an R2R3-MYB which have been reported to control anthocyanin biosynthesis in bean tissue and was proposed as a candidate for seed coat color [29]. This may be a candidate for the red kidney (*Rk*) gene and has a sequence approximately 218 kb distance from the *B* gene. 

Phvul.003G146900 also plays a role in the phenylpropanoid pathway related to seed coat color and was identified as the closest gene to the *Z* (zonal partly colored seed coat) locus in bean [30]. It is within a 100 kb window of SNPs at 35,695,104 bp associated with a*, C*, and H° exterior pod color in G19833 (Table S1). Phvul.003G203900 was associated with SNPs at 43,010,181–43,092,849 bp for the H° pod exterior in G19833. The gene is also an R2R3-MYB and may regulate color in common bean and cranberry bean seeds [29,31] (Table 6 and Table S1). 

*J* (formerly dominant *L*) was previously identified on Pv10. Researchers determined the location using RAPD markers (OL4S525 and OJ17700, respectively) and these markers were identified as occupying a region of 41,443,673–41,443,694 bp [4,20]. We identified a SNP for b* (pod exterior) at 41,403,581 bp on Pv10 in the G19833 reference genome and based on this information, Phvul.010G132400 and Phvul.010G132500 could be candidate genes for *J* (Table S1). These two candidate genes underlie non-darkening seed coat color (*jj*) in pinto and cranberry beans [32,33]. Phvul.010G132400 encodes for an SNF7 family vacuole sorting protein that in the case of *J*, could be involved in vacuolar transport of flavonoids. *J* has not been previously identified with altering colors in plant parts other than seeds. In addition to inducing a non-darkening phenotype in seeds, *jj* also diminishes flavonol and anthocyanin-based expression. It might have the same effect on these compounds in vegetative tissues, but the effect may be subtle. We were unable to phenotype seeds of the SnAP for *J* because the white seed conditioned by *p* is epistatic to *J* and most accessions in the population are white seeded.

### 4.2. Candidate Genes Associated with Photosynthetic Pathways

*Y* gene conferring yellow (wax) pod color has been mapped to Pv02 [34] (Table 1). Phvul.002G004400 and Phvul.002G006200 have been proposed as candidates for *y* [15,16]. The former encodes a pentatricopeptide repeat protein and the latter the SUF family Fe-S cluster assembly protein SufD. Pentatricopeptide repeat proteins can affect chloroplast assembly whereas SUF family genes may have a role in chlorophyll synthesis. Phvul.002G004400 was not found within a 100 kb window in both reference genomes in our study, however, in the former study [15], the candidate gene search was conducted in a 350 kb window, and if our window is expanded to this distance, this gene could be considered as a candidate for gene y. Phvul.002G006200 associated with H° pod exterior was found in the 5-593 reference genome as Pv5-593.02G005900 (SNP position = 1,468,533 bp, Table S1). Twelve other candidate genes in the region (Phvul.002G005600–Phvul.002G007100) were considered as possible candidates for *y* identified by Yang et al. [16] before ultimately selecting Phvul.002G006200. These corresponded to candidates Pv5-593.02G005300–Pv5-593.02G006800 found in the 5-593 reference genome. Additional possible candidates identified in the 200 K window bracketing the SNPs (1,620,607–1,631,424 bp) of interest were Phvul.002G014700 and Phvul.002G014800. These were candidates for b* (blue-yellow color) exterior pod color in G19833. These may actually be a single gene model that encodes an Isoflavone 2′-hydroxylase that produces a yellow pigment in the plants [35].

Phvul.002G007200 (Pv5-593.02G006900) is another potential candidate for *y* which encodes for a peptidase protein and has a hydrolase function (Table S1). Chlorophyllase, which possibly plays a role in chlorophyll degradation during photosystem II (PSII) associated turnover and fruit ripening (but not senescence), is a common hydrolase enzyme in plants. *Chlase* or CLH is located in developing chloroplast rather than in mature ones and it helps to protect the leaves from photodamage where chlorophyll turnover is necessary for PSII repair [36]. Chlase was observed in both green and non-green tissues, but its activity was higher in non-green tissues than in green tissues [37]. 

The stay-green trait found in many plant species is classified into cosmetic and functional types. Plants with the functional stay-green genes possess a photosynthesis period longer than normal, while plants with the cosmetic stay green gene remain green but lose their photosynthetic capacity during senescence. Cosmetic stay-green genes play a role in the chlorophyll catabolic pathway [38,39]. The cosmetic stay-green trait in common bean is *persistent color* (*pc*) and some snap bean cultivars have been bred to express *pc* because it imparts a more uniform and attractive pod color. Twenty-six accessions in the SnAP carry *pc*. A candidate gene for *pc* is Phvul.002G153100 (Pv5-593.02G149800) which colocalized with SNPs at 30,590,620 bp and 31,120,266 bp, respectively. These SNPs were associated with b*, C*, and H° pod exterior (Table S1). The candidate gene encodes for NYE-1, a Mg-dechelatase controlling the stay-green trait in several plant species. Our previous results based on phenotypic characterization showed that *pc* types have pod colors that are significantly different from non-*pc* types for C* pod exterior [25]. Even though it was not significantly different for H° from other green colors, *pc* had the highest H° among these cultivars. The common link between C* and H° is that both are calculated using b*.

Pv5-593.02G134200 was associated with an SNP at 29,034,300 bp and encodes a protein curvature thylakoid 1D (CURT1) and plays a role in chlorophyll and photosynthesis (PSII) accumulation during de-etiolation, shaping chloroplast and thylakoid membranes [40]. Pv5-593.02G134900 was also associated with this SNP and is a kinesin-like protein for actin-based chloroplast movement 1 (KAC1) that is affected by blue light and plays a role in chloroplast photorelocation movement that is important for photosynthesis [41] (Table 6 and Table S1).

Phvul.007G103400 is a candidate associated with a SNP at 11,974,591 bp for L* pod interior in G19833 and it is a homolog of Glyma.20G187000 (*Yl*) in *G. max*. The *Yl* gene regulates green color in plant tissues, with the mutant allele *yl* producing chlorophyll degradation, reduced PSII activity, and chloroplast structural changes [42] (Table S1).

### 4.3. Candidate Genes from Other Studies

A GWAS study focused on phenolic content used the Bean CAP Snap Bean Diversity Panel included L*a*b* pod color measurements [43]. Significant SNPs associated with L* and a* were identified on Pv02, and Pv03, and for b* on Pv05. We found SNPs associated with L*, a*, and H° pod exterior on Pv02 within the 100 kb window in the G19833 and corresponding to the same region in 5-593 (Table S1). Some significant SNPs for flower color on Pv01, Pv03, and Pv09 were also identified in that study and similar positions were found on the same chromosomes in the present study. One was associated with C* pod interior on Pv01 (48,364,135 bp), one for H° pod exterior on Pv03 (43,092,849 bp), and one for L* and b* pod exterior on Pv09 (12,097,310 bp) in the G19833 reference genome. Two SNPs for b* and C* pod exteriors on Pv03, one for L*, b*, and C* pod exterior on Pv09 in the 5-593 were also found (Table S1). Also, Phvul.009G069401 and Phvul.009G069500 associated with a SNP at 12,097,310 bp, and Phvul.011G212600 and Phvul.011G212800 associated with a SNP at 53,248,080 bp all encode for NAD(P)-binding Rossmann-fold superfamily protein, which may be candidate genes for total phenolic content associated with L* and b* pod exterior in the G19833 reference genome in our study (Table S1). In each case, the pair of gene models are adjacent to one another and may actually be a single gene model. The last two candidates were also identified as candidate genes for total seed folate content [44]. Light intensity and quality, which are necessary for photosynthesis, are very important for both folate and phenolic synthesis. Synthesis of these two compounds increases in young organs when light is sufficient [45]. 

Common bean pod color was analyzed in a Spanish Diversity Panel and significant SNPs were identified on every chromosome except Pv01 and Pv05 [35]. The authors found significant QTLs for L* and a* on Pv02 at similar position ranges (790,825–807,178 bp) where we found significant SNPs for L*, a*, and H° pod exterior and interior, and b* and C* pod interior (Table S1). Another SNP was identified on Pv06 in the Spanish study, and we found it to be associated with C* interior (G19833) in our study. Two QTLs underlying L* and b* on Pv10 were identified in their study, and associated SNPs were proposed. We found these same SNPs for a* and H° pod interior in the G19833 genome at 5,817,088 bp and H° pod interior in the 5-593 genome in our study for the QTL at 7,701,862 bp. The second QTL at 43,424,808 bp was associated with the H° pod exterior in the G19833 genome in our study. It was also associated with leaf color for L*, b*, C*, and H° (Table S1). Another SNP in the Spanish study for both the number of seeds per pod and a*was also associated with a* pod exterior in the G19833 and for L* pod interior in the 5-593 genomes, both on Pv08 in our study. The Spanish work also identified one SNP for both pod length and b* on Pv01, and two for pod length and color on Pv02. The gene on Pv01 was associated with the C* pod interior in the G19833 genome at 48,364,135 bp (Table S1). On Pv02, the first SNP was associated with b* and C* pod exterior in G19833 at 47,727,086 bp and for b*, C*, and H° pod exterior in the 5-593 genome at 48,863,854–48,940,147 bp. The second SNP was associated with C* pod exterior, and a* and H° pod interior and exterior in the G19833 at 49,401,491–49,506,969 bp, and for L* pod interior, C* pod exterior, and a* and H° pod interior and exterior in the 5-593 genome at 51,130,674–51,220,858 bp (Table S1).

Some candidate genes expressed by various traits with high phenotypic variation were found in the other studies. Phvul.001G177500 (at 43,457,300 bp) for pod exterior L* (G19833) was identified as the gene ethylene signaling (*EIN2*) that manipulated color by light regulation in pea [46], and it was a meta-QTL for Fe and Zn content, leading to light absorption on tissue and change the color, on common bean [47]. It was also a candidate for marsh spot resistance related to Mn deficiency that causes necrosis in cranberry bean seeds [48] (Table S1). Phvul.001G181000 associated with a SNP at 43,965,518 bp for b* (middle leaf) in G19833 was identified as an iron-sulfur enzyme fumarase hydratase (*FUM*) gene [49] (Table S1). This gene was also identified as Pv5-593.01G180000 at 46,993,076 bp, associated with pod exterior L* in 5-593. Phvul.001G182200 encodes UDP-glucosyl transferase and may be involved in glycosylating flavonoid molecules. It is within a 100 kb window for a SNP at 43,965,518 bp associated with leaf b* (middle) in G19833 [50]. Phvul.001G220300 (Transducin/WD40 repeat-like superfamily protein) and Phvul.001G229600 (ARM repeat superfamily protein) were candidates for seedcoat development in the darkening stage and expressed in the presence of the *P* gene in cranberry beans [31,51] (Table S1), and it is associated with a SNP at 47,592,371 bp for C* pod interior in G19833 and as Pv5-593.01G219100 (at 50,692,153–50,804,580 bp) with H° pod exterior in 5-593. 

Phvul.002G007200 (a peptidase M20/M25/M40 family protein at 790,825–807,178 bp) was identified in our study as a candidate for L*, a*, H° exterior and interior, and b* and C* interior pod color in G19833 and Pv5-593.02G006900 (at 1,586,653–1,744,283 bp) for pod exterior and interior (except b* exterior) in 5-593. It was previously identified as a candidate for bean seed shape and size [52]. Plants with light-colored and small seeds have less photosynthetic activity than plants with dark-colored and large seeds [53]. Phvul.002G021200 (encoding ASYMMETRIC LEAVES 2-like 1) and Phvul.002G021400 a homeodomain-like superfamily protein), both associated with a SNP at 2,190,426 bp in G19833. The corresponding gene models in the 5-593 genome were Pv5-593.02G020900 and Pv5-593.02G021100 (at 2,786,416–3,035,251 bp) were associated with L*, a*, H° pod interior. The former was previously found to be related to abiotic stress while the latter was related to temperature sensitivity [54,55] (Table S1). Phvul.003G203800 (histone deacetylase 6) is a member of the HDAC gene family that plays a role in plant organ development, senescence, and several biotic and abiotic stress responses [56], and was a candidate for H° pod exterior in G19833 (at 43,010,181–43,092,849 bp) (Table 6 and Table S1). Under stress conditions, both the synthesis of photosynthetic pigments and various phytochemical processes are affected [57].

Phvul.002G152700 (leucoanthocyanidin dioxygenase) was a candidate at 30,590,620 bp for b* and C* pod exterior in G19833 and it is related to delphinidin synthesis. It was downregulated in purple snap beans and was involved in the proanthocyanidin accumulation in the cranberry seed and pinto beans [31,58,59]. The corresponding candidate in 5-593 was Pv5-593.02G149400 at 31,120,266 bp for C* and H° pod exterior (Table S1). Phvul.010G132433 was a candidate associated with a SNP at 41,403,581 bp for b* pod exterior and Phvul.002G316900 (at 48,416,815 bp), Phvul.006G209500, Phvul.006G209600, and Phvul.006G209700 (at 30,376,061 bp) were all associated with SNPs for C* pod exterior in the G19833. These are all regulatory genes for anthocyanin biosynthesis [58]. 

Phvul.004G031900 and Phvul.004G032000 (at 3,910,100–3,975,292 bp) were associated with chlorosis and variation in maturity caused by a deletion in stop codon [60] and they were a candidate for C* pod interior and exterior in G19833 (the equivalent being Pv5-593.04G036100 in 5-593) (Table S1). In chlorotic tissues, as photosynthesis and the amount of chlorophyll decreases, the decrease in chlorophyll accelerates, and a color change occurs in the tissues by affecting various pigment syntheses [61].

Phvul.007G013400 (related to AP2 11) and Phvul.007G014500 (SNF1 kinase homolog 10) associated with an SNP at 941,928 bp were previously found to affect leaf chlorophyll content [62], and were associated with leaf L* (lower) in G19833. The equivalent genes in 5-593 were Pv5-593.07G012800 and Pv5-593.07G013800 at 1,296,094 bp and were associated with leaf L* (middle).

Phvul.008G019500 (MEI2-like 4) associated with a SNP at 1,633,220 bp in G19833 was a candidate for L* pod interior and a* pod exterior, and previously was identified as a candidate for flower, pod, and seed development [35,63]. Phvul.010G032700 (Sec23/Sec24 protein transport family protein) at 4,552,800–4,753,654 bp was a candidate for a*, b*, and C* pod exterior in G19833 and it was previously identified as being involved in the photosynthetic process as a response to water deficiency [63,64,65] (Table S1).

Sugars are the main source of energy for plant development and various biological processes. Variation in sugar content in pods and seeds is found in common beans, but its quantity depends on whether it is a snap or dry bean, organ size, and the cultivar. *SWEET* proteins are involved in photosynthetic carbon transport out of leaves and between cells throughout the plant [66]. Higher sugar concentration is produced by a higher rate of photosynthesis, which is associated with more chlorophyll and is expressed as darker green colors [67]. Seed coat color and sugar content are also related [5]. After flowering, the glucose and fructose fraction in pods decreases while sucrose content increases. Similarly, glucose and fructose content in seeds decreased but sucrose content did not change [68]. Phvul.002G300900 at 46,900,118 bp (Pv5-593.02G293900 at 48,119,141–48,156,962 bp), Phvul.004G017400 at 2,047,768 bp, and Phvul.009G134300 at 20,332,723–20,375,268 bp (Pv5-593.09G136600 at 22,054,215–22,086,165 bp) for C* and b* pod exterior, Phvul.006G210800 at 30,376,064 bp for C* pod exterior, Phvul.006G000600 at 281,553 bp for C* pod interior, and Phvul.008G007600 at 706,796–706,932 bp for H° pod exterior was found in our study (Table 6). These candidates were expressed in the flower, leaf, stem, and pod of the common bean where Phvul.002G300900 was downregulated by CdCl_2_ and HgCl_2_ while Phvul.004G017400 was upregulated by CdCl_2_ and NaCl [69]. Both are nodulin MtN3 family proteins. Phvul.009G134300 (Pv5-593.09G136600), also a nodulin MtN3 family protein, was identified as conditioning resistance to halo blight which is related to the salicylic acid cycle and photosynthesis because exogenous salicylic acid increases photosynthesis under water deficit which could be related to green color in common bean, and plays a role in seed coat development in cranberry bean [70,71,72].

Mineral content may affect the color of some tissues of the plant. Some candidate genes related to nutrient content were found in common beans [73], and one of them was associated with color parameters in our study. Phvul.003G001300 (tetratricopeptide repeat (TPR)-like superfamily protein) controls magnesium content and Mg deficiency can cause chlorosis in Turkish bean seed [74]. It was a candidate for b* pod exterior in G19833. Phvul.004G032300 and Phvul.008G045800 (purple acid phosphatase 23 and vacuolar ATP synthase G3, respectively) were related to phosphatase activating protein that gives dark green and purple color [75]; Phvul.004G032300 was a candidate for C* pod interior and exterior and Phvul.008G045800 was a candidate for H° pod exterior in G19833. Phvul.004G032300 corresponds to Pv5-593.04G036400 in the 5-593 reference genome. Phvul.009G127900 (NRAMP metal ion transporter 6) was related to several nutrients including Fe^2+^, Mn^2+^, Cu^2+^, and Zn^2+^ [48,76,77], and was a candidate for a* and b* pod exterior in G19833. Phvul.009G068400 (MRG family protein) was related to phosphorus use and corresponded to L* and b* pod exterior color [73]. 

## 5. Conclusions

In this study, 917 SNPs were identified for both leaf and pod tissue but only one of them in the 5-593 reference genome was found in the exact position in both leaf and pod tissues. Some of these SNPs were in the same position for leaves (in different positions in the canopy) and pods (in different environments) while some of them were found only for a specific environment and trait. The candidate genes for significant SNPs could be categorized into two groups: those varying in greenness that were associated with gene models regulating chlorophyll content and photosynthesis activity, and those affecting colors other than green that regulate the phenolic acid (flavonoid) biosynthetic pathway and expressed in flowers, pods, and seeds, and with downstream effects on abiotic and biotic stresses. Even though some candidates were found in both reference genomes, many were unique to a particular reference genome. The majority of the candidate genes associated with significant SNPs in both reference genomes were related to chloroplast proteins interacting with chlorophyll, thylakoid membranes, and photosynthesis.

## Figures and Tables

**Table 1:** Qualitative genes known to affect pod color in common bean. Candidate genes and/or linked markers with chromosome and physical positions where known are also shown. Information on genes, location and function is adapted from many sources cited in the Source column.

Gene	Chr	Function	Marker	Position (bp)	Source
*pc*	2	Encodes Mg dechelatase as the first step in the catabolism of chlorophyll	Phvul.002G153100 (v1.0 and v2.0)	29,379,137–29,381,066 (v1.0) 30,648,161–30,650,090 (v2.0)	[13]
*B*	2	Controls production of anthocyanin precursors prior to dihydrokaempferol formation	ss715644998	48,634,623–48,634,743	[14]
*y*	2	Pentatricopeptide repeat protein, or *SUF* family FE-S cluster assembly protein SufD	Phvul.002G004400, Phvul.002G006200		[15,16]
*Ace*	6	?			[17]
*V*	6	Flavonoid 3′5′ hydroxylase (F3′5′H), a P450 enzyme required for the expression of dihydromyricetin-derived flavonoids	OD12800, Phvul.006G018800	9,288,398–9,288,419	[4,18]
*P*	7	Basic helix-loop-helix protein regulating flavonoid pathway	Phvul.007G171333, Phvul.007G171466	28,752,132–28,774,743	[19]
*C*	8	MYB113 transcription factor	OAP2700, Phvul.008G038400	9,694,328–9,695,007	[4,18]
*T*	9	?	OM19400	11,731,567–11,731,944	[4]
*j* = *L*	10	Inhibits leuco-anthocyanidin production and reduces anthocyanin and flavonol production	OL4525	41,443,673–41,443,694	[4,20]
*Prp*	?	?			[21]
*arg*	?	?			[21]

Phenotypic description of qualitative genes: *pc*: persistent color, pods are uniform color and remain green at senescence; *B*: greenish-brown seed coat color, requires *P* for expression; *y*: wax (yellow) pod color; *ace*: shiny vs. dull pod epidermis; *V*: violet or black-violet anthocyanin in seeds, pods, and flowers, requires *P* for expression; *p*: white seed, flowers, and pods lacking flavonoids, *P* is required for expression of *B*, *V*, *C*, and *Prp*; *C*: complex locus affecting patterning in seeds and pods; *t*: partially colored seed coat, white flowers and no flavonoids in pods; *j*: inhibitor of expression of *t*, reduces production of flavonoids in seeds and pods, synonymous to *L*; *Prp*: controls anthocyanin expression in flowers, pods and stems, *P* and *V* required for expression; *arg*: argentum, produces greenish-gray pod color which becomes white when combined with *y*. Genes are designated as dominant or recessive, referring to the traits they express in beans.

**Table 2:** Narrow-sense heritability (%) for leaf color observed in the greenhouse in 2019 and pod colors taken from field and combined across years. Estimates are derived for two beans (And = G19833 and Mid = 5-593) reference genomes generated by an MLM (mixed linear model) in GAPIT.

	Leaf Position						Pod (Combined Years)			
	Lower		Middle		Upper		Interior		Exterior	
Trait	And	Mid	And	Mid	And	Mid	And	Mid	And	Mid
L*	28.5	30.9	30.5	31.5	29.6	28.4	80.5	80.8	78.3	77.4
a*	40.3	41.9	35.4	34.6	14.4	13.8	84.8	83.8	77.6	75.9
b*	33.4	35.4	32.9	33.0	25.6	24.8	58.1	55.9	75.0	72.5
C*	34.4	36.4	33.5	33.4	24.6	23.8	62.7	60.9	75.1	72.4
H°	31.3	34.2	29.3	30.1	26.1	25.9	83.8	83.0	74.5	72.4

L* = lightness; a* = red (+) and green (−); b* = blue (−) and yellow (+); C* = chroma (saturation); H° = hue angle (tint of color).

**Table 3:** Significant SNPs with ≥10% phenotypic variance explained (PVE) from a GWAS of the snap bean association panel (SnAP) for leaf color traits recorded in the greenhouse and mapped to Andean (G19833) and Middle American (5-593) reference genomes.

Chr	Position (bp)	−log10(*p*)	MAF	Traits	Effect	PVE (%)	H & B. P. Value
G19833							
1	43,221,928	6.99	0.09	a* (upper)	−0.61	33.64	1.94 × 10^−3^
	43,965,518	10.98	0.05	b* (middle)	2.95	30.95	2.0 × 10^−7^
	49,802,297	6.70	0.09	b* (lower)	−2.11	20.43	2.61 × 10^−3^
	49,802,297	6.13	0.09	C* (lower)	−2.07	21.47	7.07 × 10^−3^
2	33,626,429	6.25	0.07	a* (middle)	0.61	30.68	1.07 × 10^−2^
	33,626,429	6.24	0.07	C* (middle)	−1.92	13.90	1.11 × 10^−2^
7	941,928	5.95	0.06	L* (lower)	−1.40	12.12	1.08 × 10^−2^
	1,336,909	6.17	0.07	L* (upper)	−1.36	14.66	6.48 × 10^−3^
9	22,912,954	7.24	0.05	L* (upper)	1.75	24.10	1.11 × 10^−3^
	22,912,954	7.05	0.05	H° (upper)	−1.65	56.85	1.70 × 10^−3^
	23,206,537	5.81	0.05	L* (middle)	−1.42	54.93	2.94 × 10^−2^
	23,206,537	5.63	0.05	C* (middle)	−1.86	40.35	2.29 × 10^−2^
	23,206,537	9.57	0.05	b* (upper)	−3.08	55.54	5.20 × 10^−6^
	23,206,537	9.42	0.05	C* (upper)	−3.03	54.82	7.32 × 10^−6^
10	42,753,010	6.56	0.17	b* (lower)	−1.38	11.02	2.61 × 10^−3^
	42,753,010	6.49	0.17	C* (lower)	−1.39	10.66	6.25 × 10^−3^
5-593							
1	46,046,838	5.95	0.05	b* (lower)	2.35	24.72	9.90 × 10^−3^
	46,046,838	12.50	0.05	b* (middle)	3.35	31.63	8.41 × 10^−9^
	46,046,838	9.17	0.05	L* (upper)	2.17	53.42	1.79 × 10^−5^
	46,046,866	12.41	0.05	C* (middle)	−3.37	31.65	1.02 × 10^−8^
2	34,759,754	7.01	0.07	a* (middle)	0.64	28.88	2.62 × 10^−3^
3	2,905,494	8.07	0.05	b* (middle)	2.16	13.32	7.57 × 10^−5^
	2,905,494	7.91	0.05	C* (middle)	2.16	13.10	1.09 × 10^−4^
7	1,296,094	8.23	0.06	L* (middle)	1.53	16.53	1.56 × 10^−4^
9	25,192,705	7.82	0.05	a* (upper)	0.77	35.11	4.04 × 10^−4^
	25,196,015	7.57	0.05	H°(upper)	1.59	57.00	7.10 × 10^−4^
	25,196,027	7.13	0.05	L* (middle)	1.58	17.40	6.58 × 10^−4^
	25,196,027	8.20	0.05	H° (middle)	−1.82	22.67	8.28 × 10^−5^
	25,196,027	9.71	0.05	b* (upper)	2.97	33.56	5.19 × 10^−6^
	25,196,027	10.34	0.05	C* (upper)	3.23	55.11	1.21 × 10^−6^

Chr: Chromosome, MAF: Minor allele frequency, PVE: Phenotypic variance explained, H & B. P. Value: false discovery rate threshold, L*: lightness, a*: red (+)—green (−), b*: blue (−)—yellow (+), C*: Chroma (saturation), H°: hue angle (tint of color); leaf positions: lower, middle, and upper.

**Table 4:** Significant SNPs with ≥10% phenotypic variance explained (PVE) from a GWAS of the snap bean association panel (SnAP) for exterior pod color traits recorded in the field and mapped to Andean (G19833) and Middle American (5-593) reference genomes.

Chr	Year	Position (bp)	−log10(*p*)	MAF	Traits	Effect	PVE (%)	H & B. P. Value
G19833								
1	2020	43,457,300	9.33	0.06	L*	4.36	21.68	4.46 × 10^−6^
2		807,178	14.48	0.23	L*	3.46	13.91	6.37 × 10^−11^
	2019	1,991,690	13.59	0.22	a*	−1.20	26.94	2.45 × 10^−10^
	2020	1,991,690	16.40	0.22	a*	−1.50	29.88	3.82 × 10^−13^
	2 years	1,991,690	8.90	0.22	a*	−0.96	17.53	6.07 × 10^−6^
		1,991,690	10.19	0.22	C*	0.78	26.40	3.05 × 10^−7^
	2020	2,090,279	10.72	0.21	a*	4.40	18.57	6.08 × 10^−8^
	2019	2,090,300	9.78	0.21	a*	−4.51	10.77	4.56 × 10^−7^
	2 years	2,090,300	7.69	0.21	C*	3.67	21.22	4.34 × 10^−5^
		27,564,297	6.13	0.07	L*	−6.29	24.21	1.76 × 10^−3^
	2019	27,727,002	15.10	0.08	L*	4.55	11.12	7.60 × 10^−12^
	2 years	27,727,002	15.67	0.08	L*	11.06	25.78	2.05 × 10^−12^
	2019	30,590,620	21.91	0.06	b*	5.23	10.75	2.34 × 10^−18^
3	2020	10,297,623	8.63	0.14	b*	6.88	45.21	1.49 × 10^−5^
		10,888,291	10.97	0.13	b*	−1.29	28.25	1.03 × 10^−7^
		42,856,778	12.15	0.07	H°	−56.50	27.91	1.23 × 10^−9^
		43,092,849	15.52	0.06	H°	10.11	32.04	2.89 × 10^−12^
4	2020	152,406	8.72	0.05	C*	2.06	12.11	1.22 × 10^−5^
8	2 years	706,796	11.61	0.36	H°	−31.60	10.89	7.76 × 10^−9^
		706,882	15.99	0.35	H°	−6.82	22.47	6.70 × 10^−13^
		1,732,687	6.95	0.12	a*	−1.66	24.36	3.09 × 10^−4^
		1,752,346	5.64	0.24	a*	5.95	16.24	2.93 × 10^−3^
9	2020	10,765,350	6.04	0.07	C*	−1.99	11.92	2.58 × 10^−3^
10	2 years	4,715,815	9.58	0.36	b*	−0.82	10.30	1.70 × 10^−6^
11	2019	51,718,220	9.34	0.06	b*	4.20	11.76	1.09 × 10^−6^
5-593								
1	2020	46,993,079	13.37	0.05	L*	5.43	14.31	3.75 × 10^−10^
		50,939,307	27.26	0.08	H°	75.89	29.33	1.47 × 10^−23^
		51,026,803	26.04	0.07	H°	−73.80	28.52	1.21 × 10^−22^
	2 years	51,026,803	8.62	0.07	C*	1.77	19.89	2.27 × 10^−6^
2	2019	1,604,407	15.73	0.23	L*	3.08	10.18	4.93 × 10^−12^
	2020	1,604,407	18.75	0.23	L*	4.05	12.19	2.35 × 10^−15^
	2019	23,372,981	8.08	0.10	a*	−3.07	13.50	2.21 × 10^−5^
		23,874,856	20.54	0.08	H°	21.39	16.29	2.55 × 10^−17^
	2020	23,874,856	8.78	0.08	b*	−1.42	15.70	8.81 × 10^−6^
	2019	23,874,857	8.24	0.08	a*	−3.36	15.43	1.69 × 10^−5^
	2020	23,874,857	7.99	0.08	a*	−3.11	36.69	8.25 × 10^−4^
	2 years	23,874,857	7.89	0.08	a*	−3.17	15.12	6.88 × 10^−5^
	2020	25,183,895	6.81	0.10	a*	3.21	14.55	8.40 × 10^−4^
		29,034,302	12.47	0.06	H°	99.32	11.30	2.56 × 10^−10^
	2019	31,662,002	7.15	0.05	L*	4.17	13.70	3.58 × 10^−4^
	2020	31,662,002	19.09	0.05	L*	6.21	19.57	2.17 × 10^−15^
	2 years	31,662,002	17.07	0.05	L*	5.59	16.50	1.13 × 10^−13^
		31,662,002	24.98	0.05	H°	−58.80	23.82	9.17 × 10^−22^
		31,744,956	18.80	0.05	H°	−44.37	11.30	1.04 × 10^−15^
3	2019	36,729,340	30.11	0.08	b*	8.36	29.56	2.04 × 10^−26^
		36,729,340	45.77	0.08	H°	−47.89	23.78	4.50 × 10^−42^
	2020	36,729,340	10.75	0.08	b*	5.82	16.37	1.56 × 10^−7^
		36,729,340	21.31	0.08	C*	7.12	24.58	6.44 × 10^−18^
	2 years	36,729,340	24.60	0.08	b*	7.78	42.38	6.72 × 10^−21^
		36,729,340	57.34	0.08	H°	−46.96	22.01	1.22 × 10^−53^
	2019	36,757,348	28.35	0.10	b*	−7.39	20.16	5.97 × 10^−25^
		36,757,348	38.52	0.10	H°	39.62	20.75	4.04 × 10^−35^
	2020	36,757,348	10.57	0.10	b*	−4.87	17.79	1.77 × 10^−7^
		36,757,348	23.78	0.10	C*	−6.96	13.82	4.41 × 10^−20^
	2 years	36,757,348	24.23	0.10	b*	−7.39	28.44	7.79 × 10^−21^
		36,757,348	43.30	0.10	H°	36.47	13.64	6.63 × 10^−40^
4	2019	51,719,381	9.86	0.07	a*	4.04	14.47	1.23 × 10^−6^
	2 years	51,719,381	6.78	0.07	a*	3.18	19.24	6.28 × 10^−4^

Chr: Chromosome, MAF: Minor allele frequency, PVE: Phenotypic variance explained, H & B. P. Value: false discovery rate threshold, L*: lightness, a*: red (+)—green (−), b*: blue (−)—yellow (+), C*: Chroma (saturation), H°: hue angle (tint of color).

**Table 5:** Significant SNPs with ≥10% phenotypic variance explained (PVE) from a GWAS of the snap bean association panel (SnAP) for interior pod color traits recorded in the field and mapped to Andean (G19833) and Middle American (5-593) reference genomes.

Chr	Year	Position (bp)	−log10(*p*)	MAF	Traits	Effect	PVE (%)	H & B. P. Value
G19833								
1	2019	47,592,371	7.21	0.07	C*	2.00	13.9	3.96 × 10^−4^
	2 years	48,364,135	7.14	0.06	C*	1.64	15.2	4.62 × 10^−4^
2	2019	790,825	30.81	0.23	a*	−2.73	19.9	2.95 × 10^−27^
	2020	790,825	39.05	0.23	H°	4.28	18.1	1.69 × 10^−35^
	2 years	790,825	24.53	0.23	L*	−3.71	19.1	5.66 × 10^−21^
	2020	807,178	23.44	0.23	L*	3.94	14.0	6.88 × 10^−20^
		807,178	39.61	0.23	a*	3.10	16.1	4.70 × 10^−36^
	2 years	807,178	38.40	0.23	a*	2.94	15.0	7.67 × 10^−35^
		807,178	43.70	0.23	H°	−4.22	13.9	3.79 × 10^−40^
	2019	2,190,426	10.30	0.07	a*	2.01	11.7	4.85 × 10^−7^
	2 years	2,190,426	12.76	0.07	L*	3.29	15.9	1.68 × 10^−9^
5	2019	11,368,205	7.62	0.22	H°	0.99	19.0	1.23 × 10^−4^
6	2 years	14,661,470	7.87	0.44	b*	−0.82	23.8	2.59 × 10^−4^
	2020	14,665,606	9.89	0.44	C*	1.19	12.9	2.48 × 10^−6^
		18,684,799	5.71	0.19	C*	0.83	16.3	1.86 × 10^−2^
5-593								
2	2 years	1,587,342	16.18	0.23	C*	1.40	21.4	1.75 × 10^−12^
	2019	1,604,407	36.65	0.23	a*	2.85	15.8	5.96 × 10^−33^
		1,604,407	37.08	0.23	H°	−4.38	22.8	2.23 × 10^−33^
	2020	1,604,407	30.29	0.23	L*	4.54	13.3	1.37 × 10^−26^
		1,604,407	42.80	0.23	a*	3.28	17.8	4.19 × 10^−39^
		1,604,407	45.69	0.23	H°	−4.79	20.2	5.38 × 10^−42^
	2 years	1,604,407	23.48	0.23	L*	3.77	20.9	8.70 × 10^−20^
		1,604,407	41.27	0.23	a*	3.04	17.6	1.41 × 10^−37^
		1,604,407	46.11	0.23	H°	−4.65	18.0	2.06 × 10^−42^
	2020	2,979,708	14.51	0.07	H°	−3.23	10.6	4.07 × 10^−11^

Chr: Chromosome, MAF: Minor allele frequency, PVE: Phenotypic variance explained, H & B. P. Value: false discovery rate threshold, L*: lightness, a*: red (+)—green (−), b*: blue (−)—yellow (+), C*: Chroma (saturation), H°: hue angle (tint of color).

**Table 6:** Candidate genes for a subset of 14 significant SNPs with ≥10% phenotypic variance explained (PVE), SNP effect, and *p*-values from a GWAS of the snap bean association panel (SnAP) associated with exterior pod color traits recorded in the field and mapped to Andean (G19833) and Middle American (5-593) reference genomes.

Chr	Allele	Traits	Position (bp)	−log10(*p*)	MAF	Effect	PVE (%)	H & B. P. Value	Candidate Genes	Best.Hit
G19833										
2	C/T	L*.2 year	27,727,002	15.67	0.08	11.06	25.8	2.05 × 10^−12^	Phvul.002G132400	AT1G32100
									Phvul.002G132800	AT1G32080
3	G/A	H°.2020	42,856,778	12.15	0.07	−56.50	27.9	1.23 × 10^−9^	Phvul.003G202200	AT5G63050
									Phvul.003G202400	AT1G17100
									Phvul.003G203400	
	G/C	H°.2020	43,092,849	15.52	0.06	10.11	32.0	2.89 × 10^−12^	Phvul.003G203700	AT5G63100
									Phvul.003G203800	AT5G63110
									Phvul.003G203900	AT5G26660
									Phvul.003G204700	AT3G11330
									Phvul.003G205000	AT4G24690
									Phvul.003G205300	AT3G48250
									Phvul.003G205400	AT3G48250
									Phvul.003G205800	
									Phvul.003G206100	AT3G48270
8	G/A	H°.2 year	706,796	11.61	0.36	−31.60	10.9	7.76 × 10^−9^	Phvul.008G006600	AT5G65560
									Phvul.008G006700	AT5G38720
									Phvul.008G006800	AT2G19540
									Phvul.008G007500	AT5G16150
									Phvul.008G007600	AT2G39060
									Phvul.008G007700	AT5G16180
									Phvul.008G008400	AT1G66840
5-593										
1	G/T	H°.2020	50,939,307	27.26	0.08	75.89	29.3	1.47 × 10^−23^	Pv5-593.01G220900	AT5G26600
									Pv5-593.01G221300	AT1G56720
									Pv5-593.01G221400	
									Pv5-593.01G221500	AT1G09430
									Pv5-593.01G221600	AT3G07100
									Pv5-593.01G221800	AT1G56700
									Pv5-593.01G221900	AT1G09420
									Pv5-593.01G222100	AT1G09390
									Pv5-593.01G222400	AT1G09340
									Pv5-593.01G222600	AT1G56590
									Pv5-593.01G222700	AT4G15510
	A/G	H°.2020	51,026,803	26.04	0.07	−73.80	28.5	1.21 × 10^−22^	Pv5-593.01G221400	
									Pv5-593.01G221500	AT1G09430
									Pv5-593.01G221600	AT3G07100
									Pv5-593.01G221800	AT1G56700
									Pv5-593.01G221900	AT1G09420
									Pv5-593.01G222100	AT1G09390
									Pv5-593.01G222400	AT1G09340
									Pv5-593.01G222600	AT1G56590
									Pv5-593.01G222700	AT4G15510
									Pv5-593.01G223300	AT1G56500
2	T/G	H°.2019	23,874,856	20.54	0.08	21.39	16.3	2.55 × 10^−17^	Pv5-593.02G108100	AT5G66280
									Pv5-593.02G108300	AT1G68920
	A/T	H°.2020	29,034,302	12.47	0.06	99.32	11.3	2.56 × 10^−10^	Pv5-593.02G134200	AT4G38100
									Pv5-593.02G134400	AT2G22500
									Pv5-593.02G134700	AT2G22530
									Pv5-593.02G134900	AT5G10470
	G/C	H°.2 year	31,662,002	24.98	0.05	−58.80	23.8	9.17 × 10^−22^	Pv5-593.02G153100	AT1G63430
									Pv5-593.02G153300	AT1G63440
									Pv5-593.02G153400	
									Pv5-593.02G154000	AT1G63490
									Pv5-593.02G154100	
	G/T	H°.2 year	31,744,956	18.80	0.05	−44.37	11.3	1.04 × 10^−15^	Pv5-593.02G153100	AT1G63430
									Pv5-593.02G153300	AT1G63440
									Pv5-593.02G153400	
									Pv5-593.02G154000	AT1G63490
									Pv5-593.02G154100	
									Pv5-593.02G155000	AT5G41380
3	T/C	H°.2019	36,729,340	45.77	0.08	−47.89	23.8	4.50 × 10^−42^	Pv5-593.03G141800	AT2G18850
	T/C	H°.2 year	36,729,340	57.34	0.08	−46.96	22.0	1.22 × 10^−53^	Pv5-593.03G142100	AT5G57490
	T/C	H°.2019	36,757,348	38.52	0.10	39.62	20.8	4.04 × 10^−35^	Pv5-593.03G142300	AT5G57510
	T/C	H°.2 year	36,757,348	43.30	0.10	36.47	13.6	6.63 × 10^−40^	Pv5-593.03G142400	AT1G51680
									Pv5-593.03G142500	
									Pv5-593.03G142800	
									Pv5-593.03G142900	AT5G57560
									Pv5-593.03G143000	AT5G57560

Chr: Chromosome, MAF: Minor allele frequency, PVE: Phenotypic variance explained, H & B. P. Value: FDR value, L*: lightness, H°: hue angle (tint of color), Best.hit: Best hit with Arabidopsis.

## Data Availability

SnAP passport data can be found at ScholarsArchive@OSU (https://doi.org/10.7267/dj52wd79f) (accessed on 21 July 2023).

## Supplementary Materials

The following supporting information can be downloaded at: https://www.mdpi.com/article/10.3390/genes14122234/s1, Tables S1 and S2: All significant SNPs represented by tissues and each trait with associated candidate genes and their annotation in the Arabidopsis genome.
